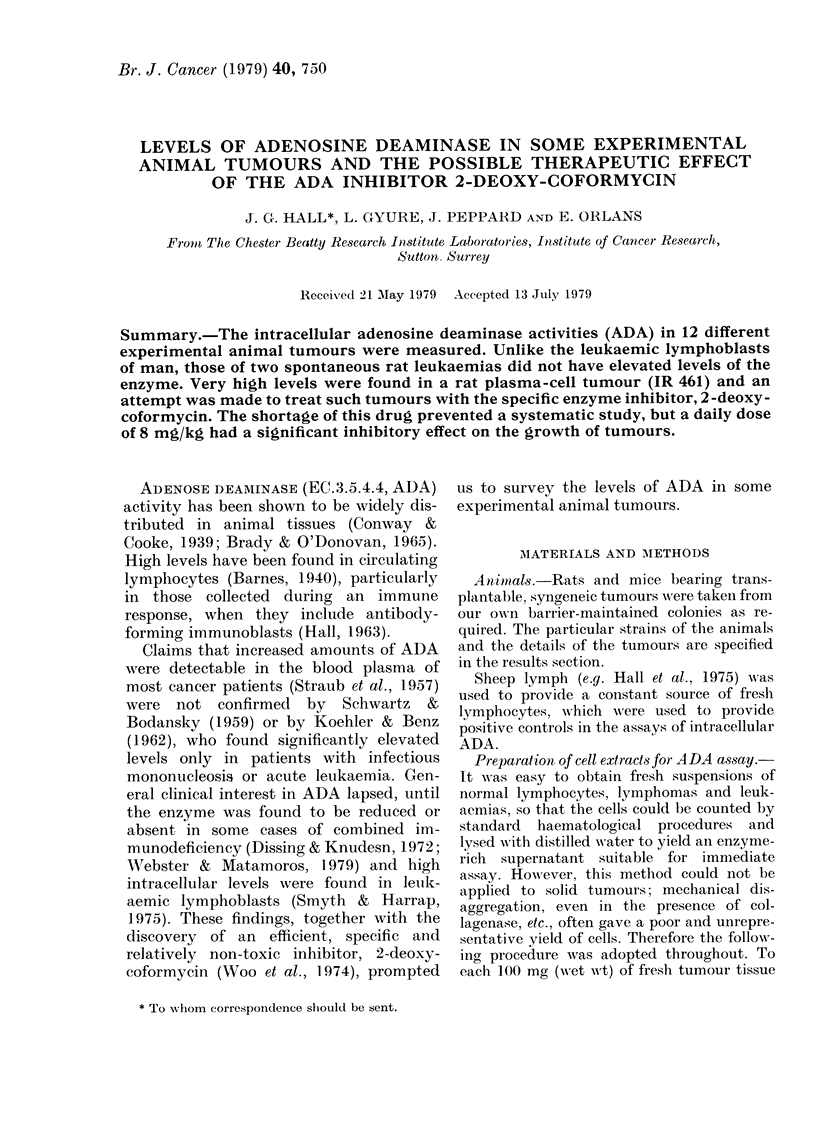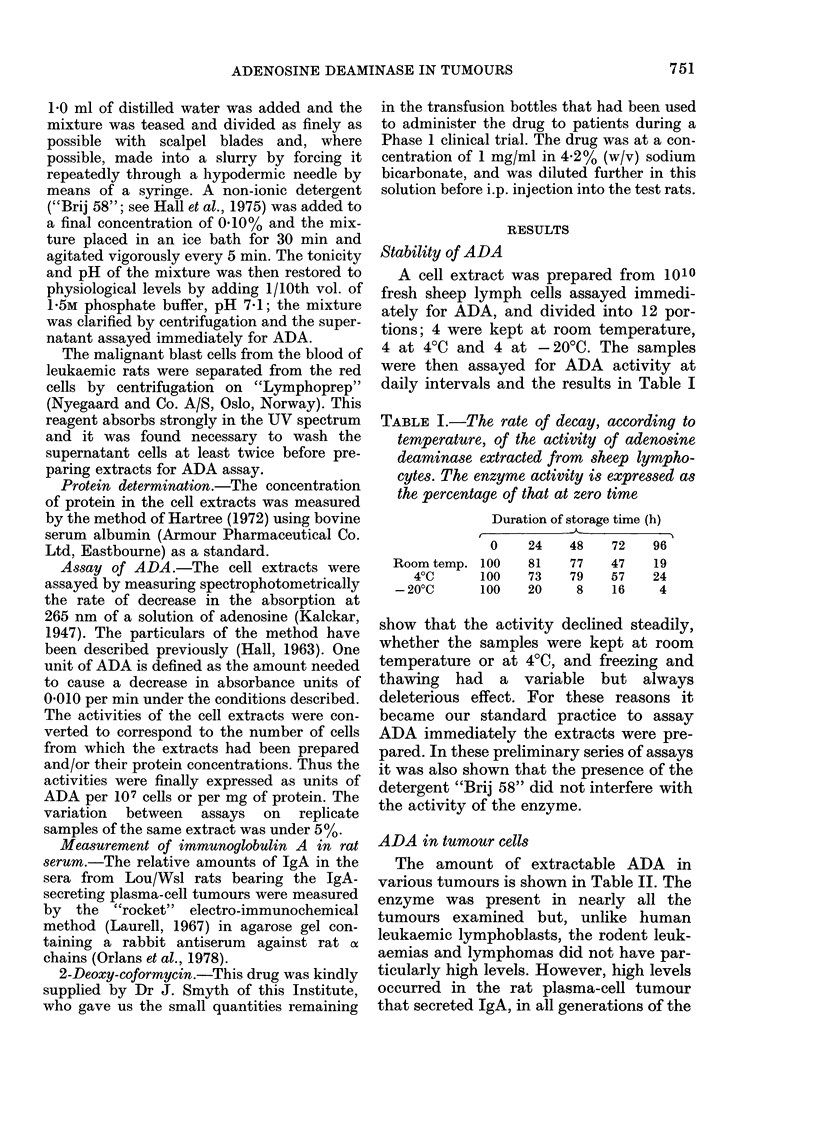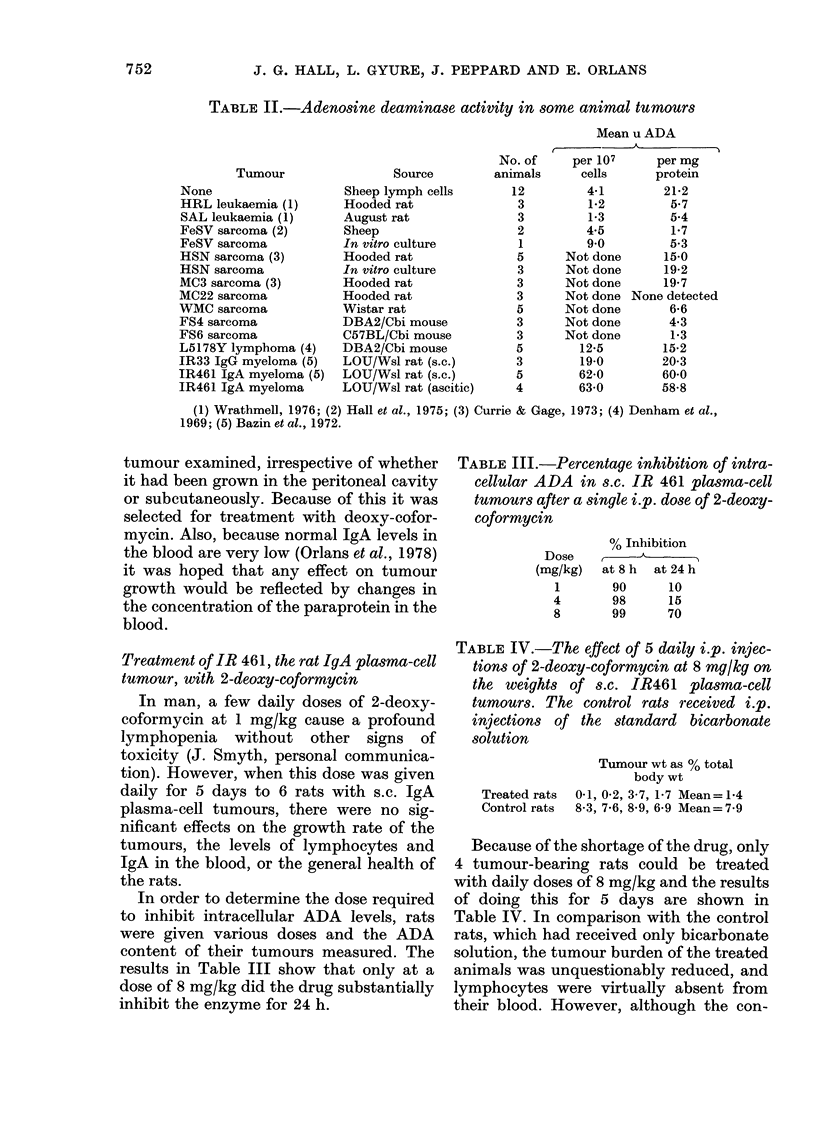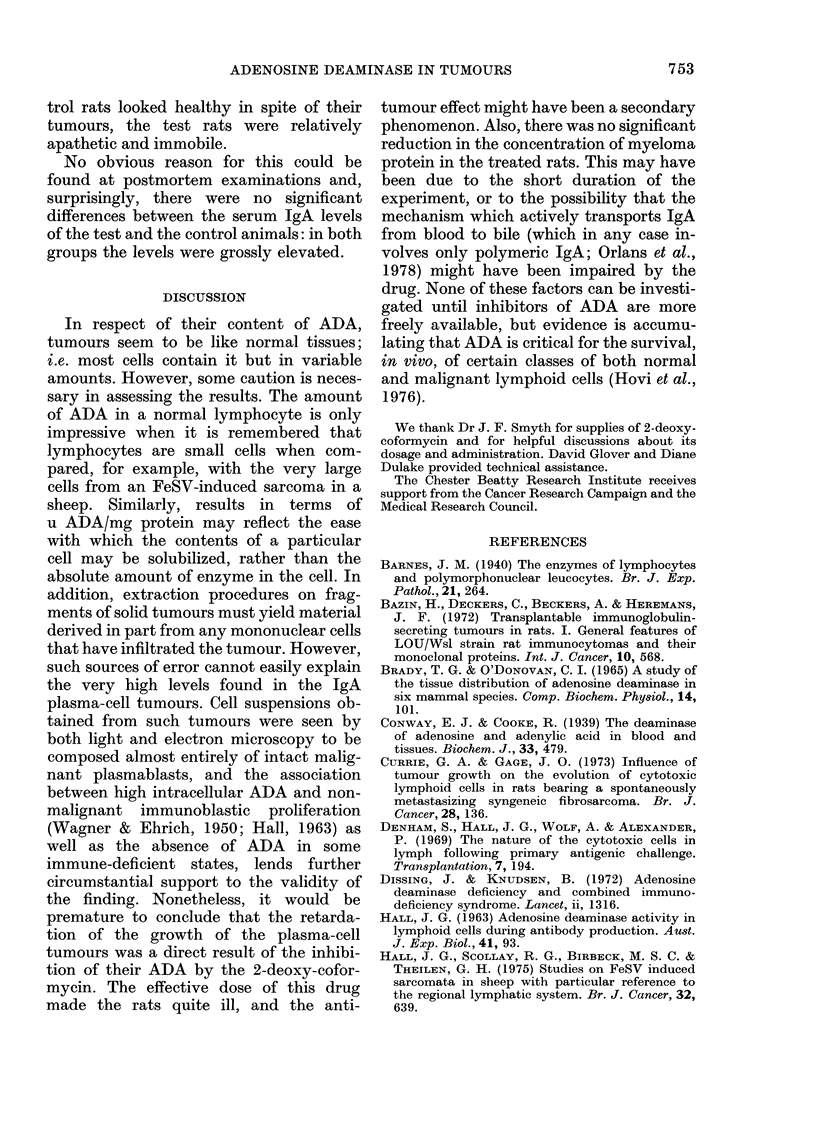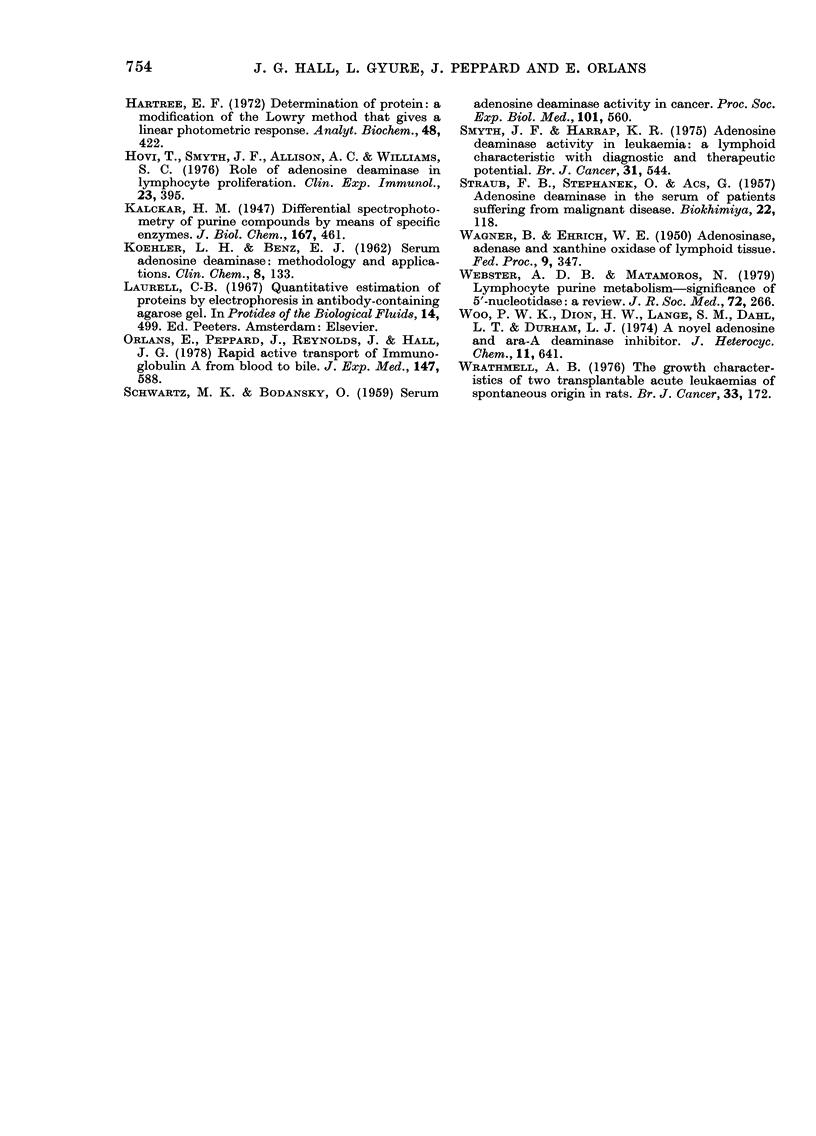# Levels of adenosine deaminase in some experimental animal tumours and the possible therapeutic effect of the ADA inhibitor 2-deoxy-coformycin.

**DOI:** 10.1038/bjc.1979.256

**Published:** 1979-11

**Authors:** J. G. Hall, L. Gyure, J. Peppard, E. Orlans

## Abstract

The intracellular adenosine deaminase activities (ADA) in 12 different experimental animal tumours were measured. Unlike the leukaemic lymphoblasts of man, those of two spontaneous rat leukaemias did not have elevated levels of the enzyme. Very high levels were found in a rat plasma-cell tumour (IR 461) and an attempt was made to treat such tumours with the specific enzyme inhibitor, 2-deoxy-coformycin. The shortage of this drug prevented a systematic study, but a daily dose of 8 mg/kg had a significant inhibitory effect on the growth of tumours.


*Br. J. Cancer* (1979) **40**, 750

### LEVELS OF ADENOSINE DEAMINASE IN SOME EXPERIMENTAL ANIMAL TUMOURS AND THE POSSIBLE THERAPEUTIC EFFECT OF THE ADA INHIBITOR 2-DEOXY-COFORMYCIN

J. G. HALL*, L. GYURE, J. PEPPARD AND E. ORLANS

*From The Chester Beatty Research Institute Laboratories, Institute of Cancer Research, Sutton. Surrey*

Received 21 May 1979 Accepted 13 July 1979

**Summary.—The intracellular adenosine deaminase activities (ADA) in 12 different experimental animal tumours were measured. Unlike the leukaemic lymphoblasts of man, those of two spontaneous rat leukaemias did not have elevated levels of the enzyme. Very high levels were found in a rat plasma-cell tumour (IR 461) and an attempt was made to treat such tumours with the specific enzyme inhibitor, 2-deoxy-coformycin. The shortage of this drug prevented a systematic study, but a daily dose of 8 mg/kg had a significant inhibitory effect on the growth of tumours.**

ADENOSE DEAMINASE (EC.3.5.4.4, ADA) activity has been shown to be widely distributed in animal tissues (Conway & Cooke, 1939; Brady & O'Donovan, 1965). High levels have been found in circulating lymphocytes (Barnes, 1940), particularly in those collected during an immune response, when they include antibody-forming immunoblasts (Hall, 1963).

Claims that increased amounts of ADA were detectable in the blood plasma of most cancer patients (Straub *et al.*, 1957) were not confirmed by Schwartz & Bodansky (1959) or by Koehler & Benz (1962), who found significantly elevated levels only in patients with infectious mononucleosis or acute leukaemia. General clinical interest in ADA lapsed, until the enzyme was found to be reduced or absent in some cases of combined immunodeficiency (Dissing & Knudesn, 1972; Webster & Matamoros, 1979) and high intracellular levels were found in leukaemic lymphoblasts (Smyth & Harrap, 1975). These findings, together with the discovery of an efficient, specific and relatively non-toxic inhibitor, 2-deoxy-coformycin (Woo *et al.*, 1974), prompted us to survey the levels of ADA in some experimental animal tumours.

### MATERIALS AND METHODS

*Animals.*—Rats and mice bearing transplantable, syngeneic tumours were taken from our own barrier-maintained colonies as required. The particular strains of the animals and the details of the tumours are specified in the results section.

Sheep lymph (*e.g.* Hall *et al.*, 1975) was used to provide a constant source of fresh lymphocytes, which were used to provide positive controls in the assays of intracellular ADA.

*Preparation of cell extracts for ADA assay.*—It was easy to obtain fresh suspensions of normal lymphocytes, lymphomas and leukaemias, so that the cells could be counted by standard haematological procedures and lysed with distilled water to yield an enzyme-rich supernatant suitable for immediate assay. However, this method could not be applied to solid tumours; mechanical disaggregation, even in the presence of collagenase, *etc.*, often gave a poor and unrepresentative yield of cells. Therefore the following procedure was adopted throughout. To each 100 mg (wet wt) of fresh tumour tissue

* To whom correspondence should be sent.

1·0 ml of distilled water was added and the mixture was teased and divided as finely as possible with scalpel blades and, where possible, made into a slurry by forcing it repeatedly through a hypodermic needle by means of a syringe. A non-ionic detergent ("Brij 58"; see Hall *et al.*, 1975) was added to a final concentration of 0·10% and the mixture placed in an ice bath for 30 min and agitated vigorously every 5 min. The tonicity and pH of the mixture was then restored to physiological levels by adding 1/10th vol. of 1·5M phosphate buffer, pH 7·1; the mixture was clarified by centrifugation and the supernatant assayed immediately for ADA.

The malignant blast cells from the blood of leukaemic rats were separated from the red cells by centrifugation on "Lymphoprep" (Nyegaard and Co. A/S, Oslo, Norway). This reagent absorbs strongly in the UV spectrum and it was found necessary to wash the supernatant cells at least twice before preparing extracts for ADA assay.

*Protein determination.*—The concentration of protein in the cell extracts was measured by the method of Hartree (1972) using bovine serum albumin (Armour Pharmaceutical Co. Ltd, Eastbourne) as a standard.

*Assay of ADA.*—The cell extracts were assayed by measuring spectrophotometrically the rate of decrease in the absorption at 265 nm of a solution of adenosine (Kalckar, 1947). The particulars of the method have been described previously (Hall, 1963). One unit of ADA is defined as the amount needed to cause a decrease in absorbance units of 0·010 per min under the conditions described. The activities of the cell extracts were converted to correspond to the number of cells from which the extracts had been prepared and/or their protein concentrations. Thus the activities were finally expressed as units of ADA per $10^7$ cells or per mg of protein. The variation between assays on replicate samples of the same extract was under 5%.

*Measurement of immunoglobulin A in rat serum.*—The relative amounts of IgA in the sera from Lou/Wsl rats bearing the IgA-secreting plasma-cell tumours were measured by the "rocket" electro-immunochemical method (Laurell, 1967) in agarose gel containing a rabbit antiserum against rat $\alpha$ chains (Orlans *et al.*, 1978).

2-*Deoxy-coformycin.*—This drug was kindly supplied by Dr J. Smyth of this Institute, who gave us the small quantities remaining in the transfusion bottles that had been used to administer the drug to patients during a Phase 1 clinical trial. The drug was at a concentration of 1 mg/ml in 4·2% (w/v) sodium bicarbonate, and was diluted further in this solution before i.p. injection into the test rats.

### RESULTS

### *Stability of ADA*

A cell extract was prepared from $10^{10}$ fresh sheep lymph cells assayed immediately for ADA, and divided into 12 portions; 4 were kept at room temperature, 4 at 4°C and 4 at −20°C. The samples were then assayed for ADA activity at daily intervals and the results in Table I

TABLE I.—*The rate of decay, according to temperature, of the activity of adenosine deaminase extracted from sheep lymphocytes. The enzyme activity is expressed as the percentage of that at zero time*

| | Duration of storage time (h) | | | | |
|---|---|---|---|---|---|
| | 0 | 24 | 48 | 72 | 96 |
| Room temp. | 100 | 81 | 77 | 47 | 19 |
| 4°C | 100 | 73 | 79 | 57 | 24 |
| −20°C | 100 | 20 | 8 | 16 | 4 |

show that the activity declined steadily, whether the samples were kept at room temperature or at 4°C, and freezing and thawing had a variable but always deleterious effect. For these reasons it became our standard practice to assay ADA immediately the extracts were prepared. In these preliminary series of assays it was also shown that the presence of the detergent "Brij 58" did not interfere with the activity of the enzyme.

### *ADA in tumour cells*

The amount of extractable ADA in various tumours is shown in Table II. The enzyme was present in nearly all the tumours examined but, unlike human leukaemic lymphoblasts, the rodent leukaemias and lymphomas did not have particularly high levels. However, high levels occurred in the rat plasma-cell tumour that secreted IgA, in all generations of the

TABLE II.—*Adenosine deaminase activity in some animal tumours*

| Tumour | Source | No. of animals | Mean u ADA per $10^7$ cells | Mean u ADA per mg protein |
|---|---|---|---|---|
| None | Sheep lymph cells | 12 | 4·1 | 21·2 |
| HRL leukaemia (1) | Hooded rat | 3 | 1·2 | 5·7 |
| SAL leukaemia (1) | August rat | 3 | 1·3 | 5·4 |
| FeSV sarcoma (2) | Sheep | 2 | 4·5 | 1·7 |
| FeSV sarcoma | *In vitro* culture | 1 | 9·0 | 5·3 |
| HSN sarcoma (3) | Hooded rat | 5 | Not done | 15·0 |
| HSN sarcoma | *In vitro* culture | 3 | Not done | 19·2 |
| MC3 sarcoma (3) | Hooded rat | 3 | Not done | 19·7 |
| MC22 sarcoma | Hooded rat | 3 | Not done | None detected |
| WMC sarcoma | Wistar rat | 5 | Not done | 6·6 |
| FS4 sarcoma | DBA2/Cbi mouse | 3 | Not done | 4·3 |
| FS6 sarcoma | C57BL/Cbi mouse | 3 | Not done | 1·3 |
| L5178Y lymphoma (4) | DBA2/Cbi mouse | 5 | 12·5 | 15·2 |
| IR33 IgG myeloma (5) | LOU/Wsl rat (s.c.) | 3 | 19·0 | 20·3 |
| IR461 IgA myeloma (5) | LOU/Wsl rat (s.c.) | 5 | 62·0 | 60·0 |
| IR461 IgA myeloma | LOU/Wsl rat (ascitic) | 4 | 63·0 | 58·8 |

(1) Wrathmell, 1976; (2) Hall *et al.*, 1975; (3) Currie & Gage, 1973; (4) Denham *et al.*, 1969; (5) Bazin *et al.*, 1972.

tumour examined, irrespective of whether it had been grown in the peritoneal cavity or subcutaneously. Because of this it was selected for treatment with deoxy-coformycin. Also, because normal IgA levels in the blood are very low (Orlans *et al.*, 1978) it was hoped that any effect on tumour growth would be reflected by changes in the concentration of the paraprotein in the blood.

*Treatment of IR* 461, *the rat IgA plasma-cell tumour, with* 2-*deoxy-coformycin*

In man, a few daily doses of 2-deoxy-coformycin at 1 mg/kg cause a profound lymphopenia without other signs of toxicity (J. Smyth, personal communication). However, when this dose was given daily for 5 days to 6 rats with s.c. IgA plasma-cell tumours, there were no significant effects on the growth rate of the tumours, the levels of lymphocytes and IgA in the blood, or the general health of the rats.

In order to determine the dose required to inhibit intracellular ADA levels, rats were given various doses and the ADA content of their tumours measured. The results in Table III show that only at a dose of 8 mg/kg did the drug substantially inhibit the enzyme for 24 h.

TABLE III.—*Percentage inhibition of intracellular ADA in s.c. IR* 461 *plasma-cell tumours after a single i.p. dose of* 2-*deoxy-coformycin*

| Dose (mg/kg) | % Inhibition at 8 h | % Inhibition at 24 h |
|---|---|---|
| 1 | 90 | 10 |
| 4 | 98 | 15 |
| 8 | 99 | 70 |

TABLE IV.—*The effect of* 5 *daily i.p. injections of* 2-*deoxy-coformycin at* 8 *mg/kg on the weights of s.c. IR*461 *plasma-cell tumours. The control rats received i.p. injections of the standard bicarbonate solution*

| | Tumour wt as % total body wt | |
|---|---|---|
| Treated rats | 0·1, 0·2, 3·7, 1·7 | Mean = 1·4 |
| Control rats | 8·3, 7·6, 8·9, 6·9 | Mean = 7·9 |

Because of the shortage of the drug, only 4 tumour-bearing rats could be treated with daily doses of 8 mg/kg and the results of doing this for 5 days are shown in Table IV. In comparison with the control rats, which had received only bicarbonate solution, the tumour burden of the treated animals was unquestionably reduced, and lymphocytes were virtually absent from their blood. However, although the control rats looked healthy in spite of their tumours, the test rats were relatively apathetic and immobile.

No obvious reason for this could be found at postmortem examinations and, surprisingly, there were no significant differences between the serum IgA levels of the test and the control animals: in both groups the levels were grossly elevated.

### DISCUSSION

In respect of their content of ADA, tumours seem to be like normal tissues; *i.e.* most cells contain it but in variable amounts. However, some caution is necessary in assessing the results. The amount of ADA in a normal lymphocyte is only impressive when it is remembered that lymphocytes are small cells when compared, for example, with the very large cells from an FeSV-induced sarcoma in a sheep. Similarly, results in terms of u ADA/mg protein may reflect the ease with which the contents of a particular cell may be solubilized, rather than the absolute amount of enzyme in the cell. In addition, extraction procedures on fragments of solid tumours must yield material derived in part from any mononuclear cells that have infiltrated the tumour. However, such sources of error cannot easily explain the very high levels found in the IgA plasma-cell tumours. Cell suspensions obtained from such tumours were seen by both light and electron microscopy to be composed almost entirely of intact malignant plasmablasts, and the association between high intracellular ADA and non-malignant immunoblastic proliferation (Wagner & Ehrich, 1950; Hall, 1963) as well as the absence of ADA in some immune-deficient states, lends further circumstantial support to the validity of the finding. Nonetheless, it would be premature to conclude that the retardation of the growth of the plasma-cell tumours was a direct result of the inhibition of their ADA by the 2-deoxy-coformycin. The effective dose of this drug made the rats quite ill, and the anti-tumour effect might have been a secondary phenomenon. Also, there was no significant reduction in the concentration of myeloma protein in the treated rats. This may have been due to the short duration of the experiment, or to the possibility that the mechanism which actively transports IgA from blood to bile (which in any case involves only polymeric IgA; Orlans *et al.*, 1978) might have been impaired by the drug. None of these factors can be investigated until inhibitors of ADA are more freely available, but evidence is accumulating that ADA is critical for the survival, *in vivo*, of certain classes of both normal and malignant lymphoid cells (Hovi *et al.*, 1976).

We thank Dr J. F. Smyth for supplies of 2-deoxy-coformycin and for helpful discussions about its dosage and administration. David Glover and Diane Dulake provided technical assistance.

The Chester Beatty Research Institute receives support from the Cancer Research Campaign and the Medical Research Council.

### REFERENCES

Barnes, J. M. (1940) The enzymes of lymphocytes and polymorphonuclear leucocytes. *Br. J. Exp. Pathol.*, **21**, 264.

Bazin, H., Deckers, C., Beckers, A. & Heremans, J. F. (1972) Transplantable immunoglobulin-secreting tumours in rats. I. General features of LOU/Wsl strain rat immunocytomas and their monoclonal proteins. *Int. J. Cancer*, **10**, 568.

Brady, T. G. & O'Donovan, C. I. (1965) A study of the tissue distribution of adenosine deaminase in six mammal species. *Comp. Biochem. Physiol.*, **14**, 101.

Conway, E. J. & Cooke, R. (1939) The deaminase of adenosine and adenylic acid in blood and tissues. *Biochem. J.*, **33**, 479.

Currie, G. A. & Gage, J. O. (1973) Influence of tumour growth on the evolution of cytotoxic lymphoid cells in rats bearing a spontaneously metastasizing syngeneic fibrosarcoma. *Br. J. Cancer*, **28**, 136.

Denham, S., Hall, J. G., Wolf, A. & Alexander, P. (1969) The nature of the cytotoxic cells in lymph following primary antigenic challenge. *Transplantation*, **7**, 194.

Dissing, J. & Knudsen, B. (1972) Adenosine deaminase deficiency and combined immuno-deficiency syndrome. *Lancet*, ii, 1316.

Hall, J. G. (1963) Adenosine deaminase activity in lymphoid cells during antibody production. *Aust. J. Exp. Biol.*, **41**, 93.

Hall, J. G., Scollay, R. G., Birbeck, M. S. C. & Theilen, G. H. (1975) Studies on FeSV induced sarcomata in sheep with particular reference to the regional lymphatic system. *Br. J. Cancer*, **32**, 639.

HARTREE, E. F. (1972) Determination of protein: a modification of the Lowry method that gives a linear photometric response. *Analyt. Biochem.*, **48**, 422.

HOVI, T., SMYTH, J. F., ALLISON, A. C. & WILLIAMS, S. C. (1976) Role of adenosine deaminase in lymphocyte proliferation. *Clin. Exp. Immunol.*, **23**, 395.

KALCKAR, H. M. (1947) Differential spectrophotometry of purine compounds by means of specific enzymes. *J. Biol. Chem.*, **167**, 461.

KOEHLER, L. H. & BENZ, E. J. (1962) Serum adenosine deaminase: methodology and applications. *Clin. Chem.*, **8**, 133.

LAURELL, C-B. (1967) Quantitative estimation of proteins by electrophoresis in antibody-containing agarose gel. In *Protides of the Biological Fluids*, **14**, 499. Ed. Peeters. Amsterdam: Elsevier.

ORLANS, E., PEPPARD, J., REYNOLDS, J. & HALL, J. G. (1978) Rapid active transport of Immunoglobulin A from blood to bile. *J. Exp. Med.*, **147**, 588.

SCHWARTZ, M. K. & BODANSKY, O. (1959) Serum adenosine deaminase activity in cancer. *Proc. Soc. Exp. Biol. Med.*, **101**, 560.

SMYTH, J. F. & HARRAP, K. R. (1975) Adenosine deaminase activity in leukaemia: a lymphoid characteristic with diagnostic and therapeutic potential. *Br. J. Cancer*, **31**, 544.

STRAUB, F. B., STEPHANEK, O. & ACS, G. (1957) Adenosine deaminase in the serum of patients suffering from malignant disease. *Biokhimiya*, **22**, 118.

WAGNER, B. & EHRICH, W. E. (1950) Adenosinase, adenase and xanthine oxidase of lymphoid tissue. *Fed. Proc.*, **9**, 347.

WEBSTER, A. D. B. & MATAMOROS, N. (1979) Lymphocyte purine metabolism—significance of 5′-nucleotidase: a review. *J. R. Soc. Med.*, **72**, 266.

WOO, P. W. K., DION, H. W., LANGE, S. M., DAHL, L. T. & DURHAM, L. J. (1974) A novel adenosine and ara-A deaminase inhibitor. *J. Heterocyc. Chem.*, **11**, 641.

WRATHMELL, A. B. (1976) The growth characteristics of two transplantable acute leukaemias of spontaneous origin in rats. *Br. J. Cancer*, **33**, 172.